# SOX2 and Bcl-2 as a Novel Prognostic Value in Hepatocellular Carcinoma Progression

**DOI:** 10.3390/curroncol28040264

**Published:** 2021-08-09

**Authors:** Zahra Hosseini-khah, Mohammad Reza Babaei, Mohsen Tehrani, Magali Cucchiarini, Henning Madry, Abolghasem Ajami, Nasser Rakhshani, Alireza Rafiei, Behrooz Nikbin

**Affiliations:** 1 Diabetes Research Center, Mazandaran University of Medical Sciences, Sari 48166-33131, Iran; z.hosseinikhah@mazums.ac.ir; 2 Department of Molecular Medicine, School of Advanced Technologies in Medicine, Tehran University of Medical Science, Tehran 55469-14177, Iran; 3 Department of Interventional Radiology, Firoozgar Hospital, Iran University of Medical Sciences, Tehran 15937-47811, Iran; babaei.mr@iums.ac.ir; 4 Gastrointestinal Cancer Research Center, Mazandaran University of Medical Sciences, Sari 48166-33131, Iran; mtehrani@mazums.ac.ir; 5 Department of Immunology, School of Medicine, Mazandaran University of Medical Sciences, Sari 48471-91628, Iran; a.ajami@mazums.ac.ir; 6 Center of Experimental Orthopedics, Saarland University Medical Center, Kirrbergerstr. Bldg 37, D-66421 Homburg, Germany; magali.madry@uks.eu (M.C.); henning.madry@uks.eu (H.M.); 7 Molecular and Cell Biology Research Center, Mazandaran University of Medical Sciences, Sari 48471-91628, Iran; 8 Gastrointestinal and Liver Diseases Research Center, Firoozgar Hospital, Iran University of Medical Sciences, Tehran 15937-47811, Iran; Rakhshani.n@iums.ac.ir; 9 Department of Immunology, Tehran University of Medical Sciences, Tehran 14177-55469, Iran

**Keywords:** SRY-box 2 (SOX2), cancer stem cell, hepatocellular carcinoma, Bcl-2, Bax

## Abstract

Sex-determining region Y-box 2 (SOX2) is a stem cell transcription factor and a major regulator of self-renewal and pluripotency of cancer stem cells (CSCs). In many types of cancer, SOX2 is dysregulated due to overexpression associated with tumor progression and low survival rate. Many HCC cases encounter recurrence and metastasis which might be due to CSCs and also apoptosis. Since little is known about the expression pattern of SOX2 and apoptotic genes in HCC, we aimed to determine the prognostic significance of SOX2, Bax, and Bcl-2 in clinicopathological features, tumor progression, and survival rate of the HCC patients. The expression of SOX2, Bax, and Bcl-2 were evaluated using qRT-PCR in 53 formalin-fixed, paraffin-embedded tissues (FFPE) of patients and 44 controls. Correlation of these genes was analyzed with clinicopathological features and tumor progression. The correlationship between SOX2 expression and ALBI grade as prognostic indicators were calculated. Survival rates were determined by Kaplan–Meier survival curves. SOX2 and Bcl-2 were remarkably overexpressed in HCC patients compared to controls (*p* = 0.04 and *p* = 0.003, respectively). A significant association was found for both SOX2 and Bcl-2 overexpression with TNM staging (*p* = 0.02, *p* = 0.04) and tumor grading (*p* = 0.01, *p* = 0.003), respectively. A significant correlation was observed: patients with SOX2 overexpression had a lower 5-year overall survival rate (*p* = 0.04); however, there was no significant association between Bcl-2 and survival (*p* = 0.5). Collectively, overexpression of SOX2 and Bcl-2, alone or combined, may be a potential marker to evaluate prognosis and response to HCC treatment.

## 1. Introduction

Hepatocellular carcinoma (HCC) is the fifth common malignancy and the third cancer-leading death in the world [1]. The five-year survival rate of HCC is about 7% and the annual mortality is approximately one million worldwide. Most HCC-related deaths might be due to inappropriate early diagnosis, lack of effective treatment [2], or extremely aggressive and metastatic phenotypes of the tumor [3]. Although conventional therapies, such as surgical resection, chemotherapy, radiotherapy, and multimodality therapies improve the survival rate of HCC patients, many cases encounter recurrence, intrahepatic spread, and extrahepatic metastasis [4]. This may be due to the presence of a small population of cancer cells with different biological characteristics, so-called cancer stem cells (CSCs). Several lines of evidence support the existence of CSCs in HCC as well as other malignancies, such as leukemias, glioblastomas, and breast, prostate, gastric, lung, and colon cancers [5]. Based on the CSC hypothesis, these cells are responsible for tumor progression via self-renewal and extensive proliferation and cause poor prognosis, resistance to treatment [6], recurrence, and low survival rate [7]. It has been demonstrated that CSCs and embryonic stem cells (ESCs) share many common properties and also the same genes might be expressed in both cells. This supports the association of stemness trait with aberrant expression of ESC genes which may be related to specific cancers [8].

ESC genes, such as the sex-determining region Y-box 2 (SOX2), OCT4, Klf4, c-Myc, Nanog, and Lin28, play key roles in the reprogramming of adult differentiated somatic cells into pluripotent stem cells [6,9], where they are essential for maintaining the pluripotency and self-renewal of embryonic stem cells [10]. Among them, SOX2, OCT4, and Nanog are considerable regulators for pluripotency and maintaining the undifferentiated state of cells [9].

SOX2, an intron-less gene on the long (q) arm of chromosome 3, is the main member of the SRY-related HMG-box (SOX) gene family [3]. The products of these genes are transcription factors that bind to DNA via their HMG domain [3,11]. SOX2 plays an eminent role in organ development, morphogenesis, and specification of cell type in the embryonic period via cell proliferation, regulation of self-renewal, and maintaining pluripotency [3,8,12]. In normal adults, SOX2 expression is limited to somatic stem cells of the brain, stomach, and colon [8] via regulating neurogenesis in the developing fetal nervous system [12]. On the other hand, SOX2 is aberrantly expressed in cancer cells, particularly CSC population, and its overexpression is related to poor prognosis, invasion, and metastasis in various cancers [8,13]. SOX2 overexpression has been shown in human tumors, such as breast, ovarian, gastric, pancreatic [11], colorectal, prostate, and skin cancers, as well as osteosarcomas and glioblastomas [9,13].

Although SOX2 expression increases the stemness degree of tumor cells [12], little is known about the expression pattern of SOX2 in HCC. Therefore, understanding the molecular and regulatory mechanisms associated with tumor initiation, progression, and metastasis would have a major impact on early detection, prognosis, and development of therapeutic strategies. There is evidence that the expression of SOX2 and SOX9 is significantly associated with adverse prognostic factors and overall survival in HCC [14]. Specifically, an approach to indirectly silence SOX2 expression was capable of preventing the proliferation, migration, and invasion of HepG2 cells [15].

To the best of our knowledge, this is the first report on evaluating the possible role of SOX2 along with apoptosis genes in the development and progression of HCC. Since most human cancers showed a failure in apoptosis [16,17], we also assessed the expression of pro-genes and anti-apoptotic, Bax, and Bcl-2, respectively.

## 2. Materials and Methods

### 2.1. Patients and Tissue Samples

Fifty-three HCC patients and forty-four controls referred to Firoozgar Hospital, Tehran, Iran, were enrolled in the study. Out of the 53 patients, 32 received sorafenib and 10 received other treatment modalities, including radiofrequency (RFA), surgery, and liver transplantation. Because of their late diagnosis, patients were mainly in advanced stages with metastasis to various organs; however, 11 patients received no treatment due to intolerance to drugs or death before initiation of treatment. Because of the small sample of patients receiving treatments other than sorafenib, a five-year survival analysis was just performed for patients undergoing sorafenib alone. They received systemic therapy with sorafenib (400 mg twice daily for 24 weeks) as standard systemic therapy available. HCC was diagnosed based on the current American Association for Study of Liver Diseases (AASLD) guidelines [18], considering the clinical and imaging findings, confirmed via laboratory and pathological evaluations. Histological grading of tumor differentiation was classified according to Edmondson and Steiner [19]. The TNM staging system was based on the American Joint Committee on Cancer (AJCC) and the International Union Against Cancer (UICC) [20]. Patients with secondary liver cancer, such as metastasis from other malignancies, were excluded. The control group included 44 subjects who were candidates for liver biopsy because of raised liver enzymes, abdominal pain, portal hypertension, or hepatosplenomegaly, but finally, histopathological examinations showed no evidence of malignancy or cirrhosis. Written informed consent was obtained from all participants and the protocol of the study was approved by the Ethics Committee of Tehran University of Medical Sciences (Ethics Committee Code: IR.tums.rec.1394.388). The core needle biopsy (CNB) method was used to obtain a biopsy from liver parenchyma with portal tracts. Tissue samples were fixed in formalin 10% to preserve vital structures and embedded in a paraffin wax block. Formalin-fixed paraffin-embedded tissues (FFPET) were taken from all study subjects between October 2014 and May 2016. Four to five sections of FFPE samples were dissected with a thickness of 10 µm to enrich the tissue content for RNA isolation.

### 2.2. Total RNA Extraction and cDNA Synthesis

After deparaffinization of FFPE tissues, using xylene and ethanol, RNA extraction was accomplished using RNeasy FFPE Kit (Qiagen, Hilden, Germany) according to the manufacturer’s recommendations. DNase treatment was also performed to eliminate genomic DNA. To evaluate the quality and quantity of extracted RNA, spectrophotometry and electrophoresis on 1% agarose gels were used. Extracted RNA was prone to synthesize complementary DNA (cDNA) using AccuPower^®^CycleScript RT Premix (dN_6_) kit (Bioneer, Daejeon, South Korea) according to the manufacturer’s protocol.

### 2.3. Gene Expression Analysis Using qRT-PCR

Quantitative expression of SOX2, Bax, and Bcl-2 in FFPE tissues was performed using quantitative reverse-transcriptase polymerase chain reaction (qRT-PCR). The primers were designed using Allele ID 7.5. Table 1 shows the primer sequences of target and housekeeping genes. All tests were performed in triplicate. qRT-PCR was performed in a 25 µL reaction volume containing: 12.5 µL SYBR Premix EX TaqII (2X) (Takara, Shiga, Japan), 50 ng of total RNA input, and 50 nM primers. Gene amplifications were performed using an IQ5 multicolor real-time PCR detection system (BioRad, Hercules, CA, USA). Amplification reactions were initiated with 1 cycle at 94 °C for 2 min, followed by 40 cycles of 94 °C for 30 s; 58 °C (for SOX2), 60 °C (for Bax and Bcl-2) for 30 s, and 72 °C for 45 s. GAPDH was also used as a housekeeping gene to normalize gene expression patterns. Relative gene expression analysis was carried out using the 2^−ΔΔCT^ method (*Livak and Schmittgen, Methods, 2001*). ΔCt values were determined by subtracting the Ct value of the target gene (SOX2, Bax, or Bcl2) from the reference gene (GAPDH) and ΔΔCt values were calculated by subtracting the ΔCt value of each HCC sample from the average ΔCt value of the control samples.

### 2.4. Statistical Analysis

Statistical analyses were performed using the SPSS version 17.0 and GraphPad Prism 5 software. All data displayed a normal distribution. Quantitative variables were assessed using independent t-test or one-way analysis of variance (ANOVA). Multivariate logistic regression analysis was used to assess any association in the gene expression between patients and controls by calculating odds ratios with 95% confidence intervals. The Kaplan–Meier survival curves were used to determine the survival rate.

## 3. Results

### 3.1. Demographic and Clinical Characteristics of the Patients

A total of 97 subjects including 53 HCC patients and 44 controls were studied. The majority of patients were males (75.47%). As shown in Table 2, there was no significant difference in age (*p* = 0.06) and gender (*p* = 0.819) between the patients and the controls. Evaluation of liver enzymes showed significant differences between the two groups (*p* < 0.05). On the other hand, HCC patients had a nearly two-fold increase in AST, ALT, and ALP compared to the controls. According to Edmondson’s grading, 35.8% of tumor harboring patients were in grade I.

TNM staging showed that 60.41% of patients were in stage I (Table 3). To better evaluate the implication of SOX2 in the disease’s severity, we combined grade I with grade II patients as low-grade disease, and grade III with grade IV patients as high-grade disease. Stage I and stage II were considered early-stage diseases while stage III and stage IV were listed as advanced stages.

### 3.2. SOX2 Is Up-Regulated in HCC Along with Bcl-2

**Figure 1:**
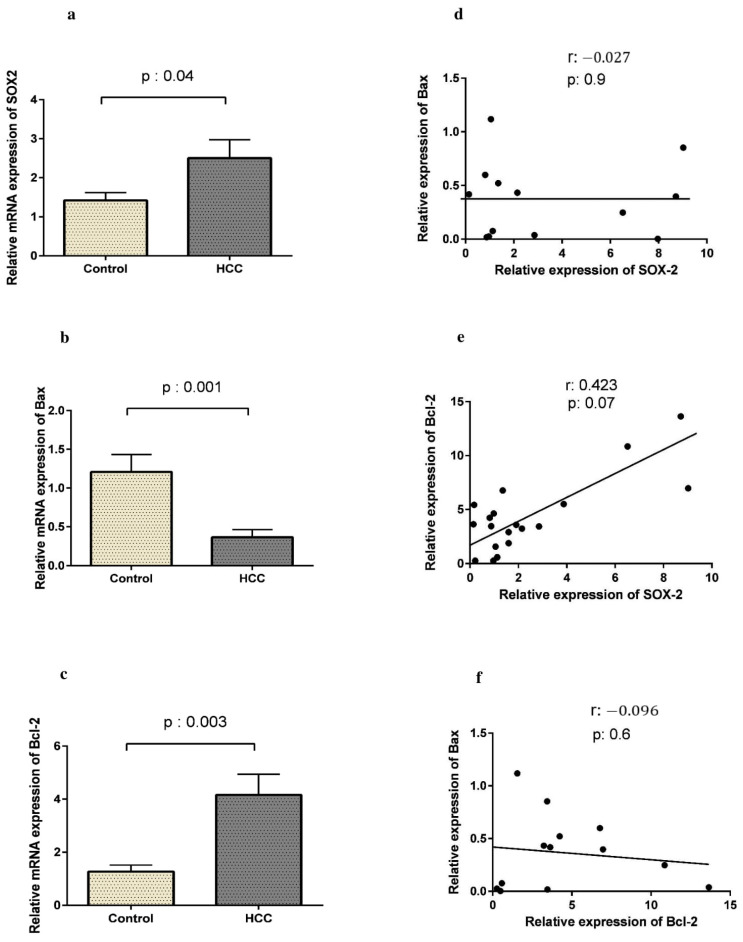
Relative expression of SOX2, Bax, and Bcl-2 in HCC patients and controls by 2^−∆∆CT^ method; qRT-PCR analysis showed an up-regulation of SOX2 (**a**) as well as down-regulation of Bax (**b**), and up-regulation of Bcl-2 in HCC patients (**c**). There was no correlation between Bax and SOX2 expression (**d**), a positive correlation was found between Bcl-2 and SOX2 expression in patients (**e**). There was no correlation between Bax and Bcl-2 expression (**f**).

Using qRT-PCR, we first showed an up-regulation of the SOX2 gene in HCC. As shown in Figure 1a, SOX2 expression was prominently higher in HCC patients than in controls (2.50 ± 2.56 vs. 1.42 ±1.02, respectively, *p* = 0.04). To analyze apoptotic activity in HCC samples, we evaluated Bax and Bcl-2 expression, as pro- and anti-apoptotic genes, respectively, in FFPE tissues from the patients and the controls. The expression of Bax was significantly lower in HCC patients compared to the controls (0.36 ± 0.34 vs. 1.20 ± 0.95, *p* = 0.001, Figure 1b), while Bcl-2 expression was higher in patients than in controls (4.16 ± 3.44 vs. 1.27 ± 0.98, *p* = 0.003, Figure 1c). Moreover, a weak positive correlation was observed between the expression of SOX2 and Bcl-2 in HCC patients (r = 0.423, *p* = 0.07). However, as Figure 1f shows, there was no significant correlation between the expression of Bax and Bcl-2 in the patients (r = −0.096, *p* = 0.6).

Since hepatitis B virus (HBV) and/or hepatitis C virus (HCV) infection is the most important risk factor for HCC establishment, we further classified HCC patients into HBV or HCV positive or negative groups. As shown in Table 4, there was no significant association between infection with HBV or HCV and the expression of SOX2, Bax, or Bcl-2 in HCC patients.

### 3.3. The Expression of SOX2, Bax, or Bcl-2 Correlates with Liver Enzyme Activity and AFP Levels in HCC Patients

**Figure 2:**
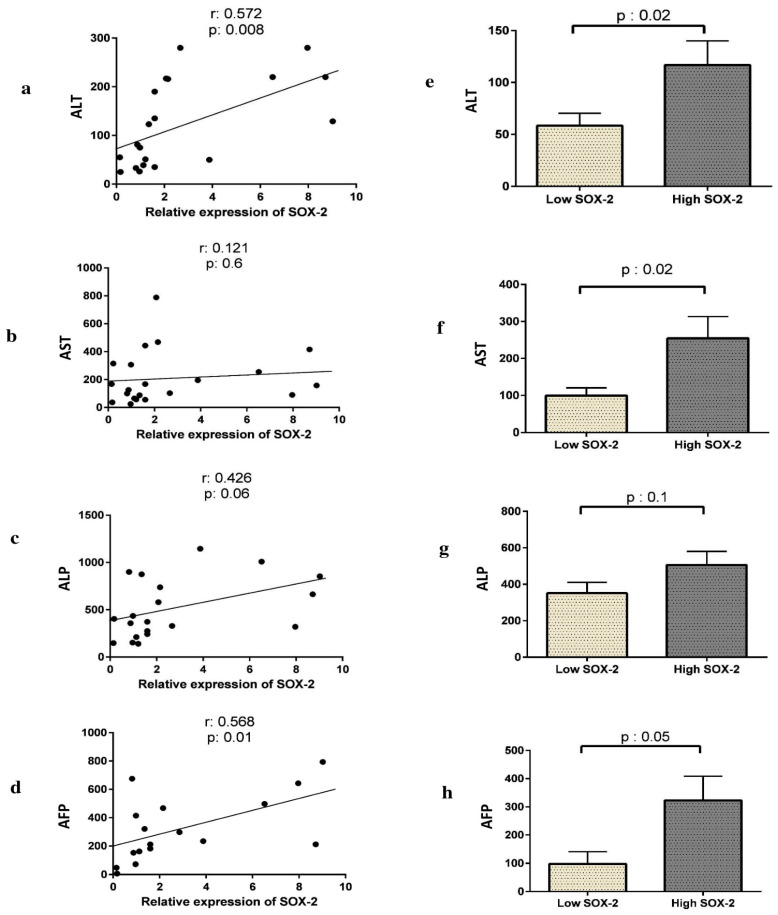
Correlation between SOX2 expression and liver function activity in HCC patients. The expression of SOX2 was positively correlated with ALT (**a**) and AFP (**d**). Correlation with AST and ALP was marginally significant (**b**,**c**). Expression of SOX2 and liver function activity in HCC patients. ALT (**e**), AST (**f**), and AFP (**h**) levels were significantly increased in high-SOX2 compared to low-SOX2 expression patients. There was no statistically significant association between SOX2 expression and ALP (**g**).

We examined the correlation between SOX2, Bax, or Bcl-2 expression with liver function tests (LFT), including AST, ALT, ALP, as well as alpha-fetoprotein (AFP) tumor marker. SOX2 expression was significantly correlated with AFP (*p* = 0.01) and ALT (*p* = 0.008), while it was not correlated with ALP and AST (Figure 2) (*p* < 0.05).

Furthermore, a significant positive correlation was shown between Bcl-2 expression and both ALT (*p* = 0.02) and ALP (*p* = 0.01), while a significant negative correlation was observed between Bax expression and AFP (*p* = 0.03) (Table 5).

We then explored any correlation between SOX2 expression pattern and liver function. As Figure 2 shows the HCC patients with high expression of SOX2 had higher AST and ALT activity compared to those with low SOX2 expression (*p* = 0.02 and *p* = 0.02, respectively). The levels of AFP were also higher in patients with SOX2 overexpression (*p* = 0.06). Moreover, as Table 4 shows, HCV-positive HCC patients had a significantly higher ALP than HCV-negative ones (*p* = 0.07).

### 3.4. SOX2 Expression Is Correlated with Tumor Progression and Lower Survival Rate

**Figure 3:**
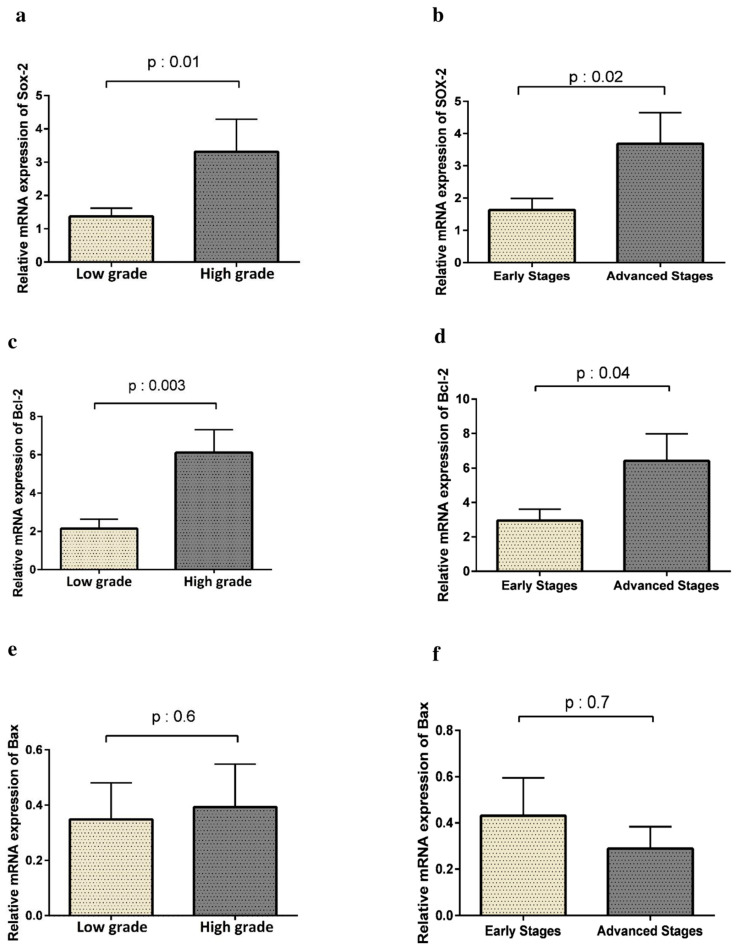
Based on the Edmondson grading system, 71.6% (*n* = 38) of tumor samples were low grade and 28.3% (*n* = 15) were high grade. Moreover, based on the TNM staging system of the AJCC/UICC, 68.75% (*n* = 33) of patients were in the early stage and 31.25% (*n* = 15) were in the advanced stage of HCC. mRNA expression of both SOX2 and Bcl2 were higher in high-grade tumors than in low-grade ones (**a**,**c**) and also higher in tumors with advanced stages than those with early stages (**b**,**d**). There was not a significant difference in Bax expression between tumors with different grades or stages (**e**,**f**).

To explore the role of SOX2 in HCC progression, the correlation of SOX2 expression was assessed with tumor grade and disease stage. As shown in Figure 3, the expression of SOX2 was significantly more prominent in high-grade HCC patients than in those with low-grade tumors (*p* = 0.01). Moreover, HCC patients in advanced stages had higher levels of SOX2 expression compared to those in the early stages (*p* = 0.02). Therefore, SOX2 overexpression was significantly correlated with HCC progression. In line with changes in SOX2 expression, higher expression levels of Bcl-2 were also coordinated with tumor grade (*p* = 0.003) and the progression of the tumor (*p* = 0.04). However, we did not find any statistically significant association between the expression of Bax and tumor grading and HCC progression (*p* < 0.05).

Subsequently, to evaluate the relationship between SOX2 expression and overall survival, HCC patients undergoing sorafenib treatment were divided into two groups based on SOX2 expression: low and high expression. The mean expression level of SOX2 in 32 patients (sorafenib-received) was considered as a cut-off point (2.431) [21].

**Figure 4:**
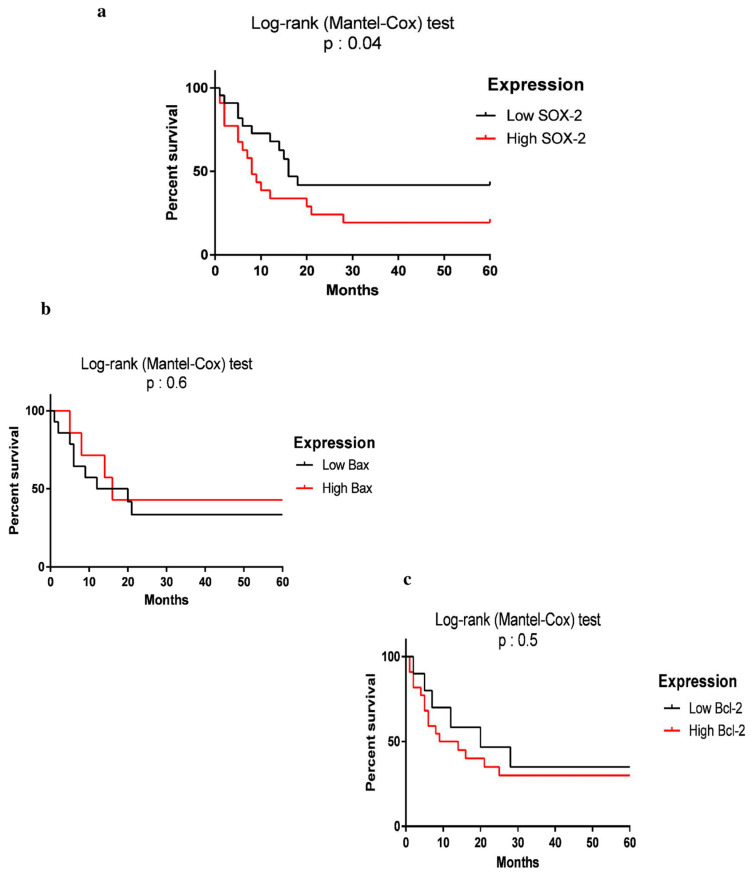
Kaplan–Meier plot for overall survival of HCC patients using the log-rank (Mantel–Cox) test; patients with higher expression of SOX2 had significantly lower 5-year survival rate compared to those with lower expression of SOX2 (log-rank: 3.082, *p* = 0.04). The median overall survival for low SOX2 was 10.5 months and for high SOX2 was 6.5 months. (**a**), Bax (log-rank: 0.1687, *p* = 0.6) and Bcl2 (log-rank: 0.3873, *p* = 0.5) expression was not correlated with the survival rate of HCC patients (**b**,**c**).

In a prospective study, we followed 32 HCC patients who had been undergoing sorafenib for 5 years. Out of 32 patients, 17 patients showed high expression and 15 patients had low SOX2 expression. OS was measured from the time of diagnosis of HCC to death or until the end of the 5-year follow-up period. As Figure 4 shows, during the follow-up period, 19.5% of the patients were alive. Patients with high SOX2 expression had a lower 5-year OS compared to those with low expression of SOX2 (*p* = 0.04). There was no significant relationship between Bax and Bcl2 expression and OS of the patients with HCC (*p* < 0.05). Analysis of the OS rate in HBV- or HCV-infected HCC showed that viral hepatitis infection did not statistically affect the survival rate of the patients (*p* = 0.8). We calculated the power of analysis using the G*Power software, and it was 0.8848983.

## 4. Discussion

This study aimed to determine the prognostic value of SOX2 in tumor progression and survival rate of patients with HCC. We found SOX2 mRNA expression in HCC tissue samples more than two folds higher than the normal tissue samples. Our results were consistent with studies showing SOX2 overexpression in gastric cancer [22], lung squamous cell carcinoma [23], gastrointestinal cancers [12], cervical cancer [24], gliomas [25], and pancreatic carcinoma [3,26].

Our results also showed that SOX2 overexpression was significantly higher in high-grade tumors compared to low-grade samples. Moreover, the SOX2 was up-regulated up to 2.5 folds in advanced stages than in the early stages of the disease. These results indicate that overexpression of SOX2 could promote oncogenic traits in HCC. The results of some studies were in line with our study; conversely, some studies have reported conflicting results. A decreased number of SOX2-expressing cells in primary benign and malignant skin tumors has been shown to lead to tumor regression, which is consistent with the vital role of SOX2 in tumor progression [27]. Moreover, a meta-analysis with 1713 patients with breast cancer reported significant associations between SOX2 expression and increased tumor size, high tumor histological grade, increased risk of lymph node metastasis, and the highly aggressive triple-negative phenotype; nevertheless, no association was observed for TNM stage [28].

Overexpression of SOX2 in MCF-7 breast cancer cells has also been shown to increase cell proliferation and tumorigenesis by upregulating cyclin D1 and subsequently facilitating G1/S cell cycle transition [29]. Moreover, an integrated analysis of the clinical and survival impact of SOX2 in colorectal cancer showed that SOX2 expression was not significantly related to clinical stage and histological grade [30].

In solid tumors, such as HCC, a minor subset of cells, called CSCs can increase the number of differentiated cells leading to the production of tumor bulks [4]. According to the CSC hypothesis (ability of self-renewal, multilineage differentiation), tumorigenicity might be correlated with CSCs [4,5]. Since SOX2 is a key marker of CSCs and regulates self-renewal and differentiation of stem cells via modifications in transcriptional, translational, and post-translational levels [31], it is likely that SOX2 transcriptionally regulates epigenetic alterations, such as histone deacetylation, necessary for the maintenance of self-renewal in liver CSC [32]. This might explain the role of SOX2 in tumor progression via the self-renewal and activation of reprogramming mechanisms of cancer cells.

SOX2 is involved in many signaling pathways such as VEGF, MAPK, Notch, P53, Wnt, and JakSTAT, and regulates the expression of many genes, which are very important regulators of CSCs and contribute to proliferation, migration, and invasion of cancer cells, thereby affecting cancer progression, prognosis, and resistance to anticancer therapies [33]. As a result, SOX2 overexpression promotes and maintains the stemness of CSCs, which confirms its role in oncogenesis and tumor progression in HCC.

There are some explanations for the contradictory results regarding the tumorigenic role of SOX2 in different cancers. The first one is ethnic variation, as different studies have been carried out in different ethnic groups, which may affect the results. An epigenetic mechanism, for example DNA methylation which can regulate gene expression, could explain racial differences in breast tumor clinical biology and outcomes. Kathleen Conway et al. suggested that racial differences in the methylation of cancer-related genes are detectable in both tumor cells and peripheral blood leukocytes from breast cancer cases [34] The other explanation is the use of different/unclear cut-off values in SOX2 expression. In other words, the cut-off point has been considered in some studies as a criterion for defining low and high expression. For example, in one study the median may be the basis for determining the cut-off point [21], or the mean or even positive or negative expression of the gene may be used for this purpose.

In addition, the size of the samples in various studies affects the results of the association between SOX2 expression and the pathological features and prognosis of HCC. Cynthia Stretch et al., who investigated the effect of sample size on differential gene expression, found that this parameter affects the results of microarray analysis, resulting in unstable gene lists and poor prediction accuracy [35]. Further studies with higher numbers of study subjects are therefore necessary to determine the correlation between SOX2 expression and tumor progression in HCC.

In order to obtain a clear illustration of the role of SOX2 during the progression of HCC, we evaluated the correlation between SOX2 expression and liver functions. Interestingly, we found a strong association between SOX2 expression and the concentrations of serum ALT, a specific enzyme of the liver, and also of the AFP tumor marker. Increased concentrations of ALT and AFP in HCC patients associated with SOX2 overexpression confirmed the role of SOX2 as a biomarker for HCC progression. Such correlation indicates that SOX2 may be considered a prognostic marker for liver damage in HCC. In accordance with our results, one study in 2019 suggested that the levels of serum AFP may effectively improve the diagnostic performance of HCC, introducing AFP as a new simple and economic indicator for clinics [36]. Moreover, another study in 2020 showed the superiority of AFP, along with ALT, AST, and NLR, as a combined biomarker system for HBV-HCC diagnosis relative to the use of each single biomarker or any other combinations in patients [37]. Further research is needed to address whether overexpression of SOX2 in HCC occurs before that of other tumor markers, in particular of AFP. The expression of the Bcl-2 family genes was evaluated in cancerous and normal liver tissues to investigate the correlation of SOX2 with apoptosis in HCC. An up-regulation of Bcl-2 and a down-regulation of Bax were found in HCC patient tissues compared to the control group. Our data were in line with previous studies in liver cancer [38,39] and also other types of cancers, such as prostate [40], ovarian [41], and lung cancers [42]. The expression of Bcl-2, an anti-apoptotic gene, mimicked the trend of SOX2 changes in HCC progression, whereas Bax expression, a pro-apoptotic gene, showed a different pattern compared to SOX2 and Bcl-2 genes. Therefore, it is likely that the overexpression of SOX2 and Bcl-2 was associated with HCC progression. Although apoptosis is reduced in most cancers, studies in HCC have reported contradictory results [43]. In one study, the Bcl-2/Bax ratios of the tumors were not different between cancerous and noncancerous liver tissues and there was no correlation between apoptosis and clinical outcomes in HCC [44]. Another study indicated that the expression of Bcl-2 decreased, while the expression of Bax increased with the development of liver cancer [16]. Finally, another study reported a decrease in expression of both Bax and Bcl-2 in HCC patients compared to controls [17].

Expression of apoptotic genes including Bax and Bcl-2 may vary at different stages of tumor development and progression, such as a hepatic lesion, proliferation/cirrhosis, and carcinogenesis. As a result, the balance of the expression of these genes might play a vital role in the progression of liver cancer [45]. It has been shown that the silencing of SOX2 caused apoptosis in some lung cancer cells, but not in noncancerous cells, such as BEAS-2B and NL-20 [42]. It has been reported that SOX2 could induce BCL2L1 expression, which rescued the cells from apoptosis and autophagy. SOX2–BCL2L1 signaling increased chemoresistance by maintaining mitochondrial integrity and enhancing cell survival during cancer progression [46]. Indeed, both apoptosis and autophagy were decreased upon SOX2 overexpression which induced BCL2L, a prosurvival regulator, and critical anti-apoptotic protein in apoptotic and autophagic pathways [47]. This supports the idea that SOX2 induces BCL2L1-mediated survival signaling and associates with chemotherapy resistance [42].

Survival analysis of the two subgroups of HCC patients, including those with high and low expression of SOX2, showed that patients with high expression of SOX2 had lower overall survival (OS) and poor prognosis compared to those with low SOX2 expression. In line with our results, several studies reported that SOX2 overexpression was correlated with poor prognosis in various cancers [11,42,48]. In this regard, Saigusa et al. evaluated the association between SOX2, CD133, and Oct4 expression, and the clinical outcomes of patients with colorectal cancer who underwent chemoradiotherapy before surgery. They demonstrated that increased expression levels of these genes were significantly associated with recurrence and poor survival rates [49]. Moreover, Lu et al. reported that patients with high expression of SOX2 in human squamous cell lung tumors and some adenocarcinomas had lower survival rates [50]. Han et al. also indicated that SOX2 positivity was associated with poor disease-free survival (DFS) and recurrence-free survival (RFS) in advanced cancer [51]. Li et al. reported the overexpression of SOX2 in the nuclei of non-small-cell lung cancer (NSCLC) cells and suggested this gene as a promising marker in the diagnosis and treatment of NSCLC [52]. Some meta-analyses also suggested the prognostic significance of SOX2 and its positive association with poor survival in human cancers [53]. Javaeed et al. in a meta-analysis showed that SOX2 overexpression was associated with shorter overall survival in patients with cancer [54]. Another meta-analysis on colorectal cancer showed SOX2 correlation with worse survival [30].

In contrast, some studies were inconsistent with our study. For example, Kim et al. evaluated OCT4 and SOX2 expression in cervical cancer samples which showed that patients with higher expression of SOX2 had better OS than those with low expression [55]. This conflict might be due to the different methodologies or differences in the studies’ sample size.

As mentioned, CSCs are self-renewal and highly proliferative, thus responsible for tumor progression, chemotherapy/radiotherapy resistance, metastasis, and recurrence [6]. Hepatic CSCs regulate a hierarchical network that involves tumor formation and they cooperate in the metastasis and prognosis of HCC [56]. In addition, overexpression of SOX2, a biomarker of putative CSCs [57], causes the durability of liver cancer cells and promotes HCC progression, and thereby decreases patient survival. Therefore, targeting CSCs has become a promising way to effectively treat cancer patients [58].

Regarding the molecular mechanisms of SOX2 in tumor invasion and metastasis, some studies have shown that SOX2 up-regulation results in increased phosphorylation of multiple pro-metastatic proteins, such as Src, FAK, and their downstream molecules, such as p130-cas. Phosphorylation of p130-cas might be the result of the Src kinase activation. The Src kinase activity is essential for SOX2-mediated adhesion and migration of cells. In this regard, it has been shown that knockdown of the SOX2 gene could lead to reduced phosphorylation of these proteins which would consequently increase cell adhesion to matrigel, fibronectin, and type I collagen, but not to laminin, and would decrease cell migration followed by increased patient survival [13].

## 5. Conclusions

Our results indicated the overexpression of SOX2 in HCC tumor tissues compared to normal tissues, and that was correlated with clinical stage and histological grade which confirms the role of CSCs in the development of HCC. Moreover, Bcl-2 was upregulated along with SOX2 and associated with high-grade tumor as well as advanced-stage of HCC. In addition, SOX2 overexpression was positively correlated with poor OS in HCC patients. These suggest SOX2 as a potential novel biomarker for prognosis and diagnosis of HCC and also as an alarm to control disease progression. Our data might also help to develop effective targeting therapy strategies, such as the silencing of SOX2 in human HCC, which would increase the apoptosis of CSCs in vitro and significantly suppress tumorigenicity in vivo. However, several questions about the gene regulatory pathways remained to be answered to disclose functional aspects of new targeted therapeutic strategies.

## Figures and Tables

**Table 1:** Sequence of the oligonucleotide primers used in qRT-PCR.

Target Gene	Sequence (5′–3′)		Accession Number	Amplicon Size (bps)
*SOX2*	Forward Reverse	TTGTCGGAGACGGAGAAG GGCAGCGTGTACTTATCC	NM_003106.4	140
*Bax*	Forward Reverse	TGGCAGCTGACATGTTTTCTGAC TCACCCAACCACCCTGGTCTT	NM_001291430.2	195
*Bcl-2*	Forward Reverse	TCGCCCTGTGGATGACTGA CAGAGACAGCCAGGAGAAATCA	NM_000633.3	134
*GAPDH*	Forward Reverse	GGTGGTCTCCTCTGACTTCAACA GTTGCTGTAGCAAATTCGTTGT	NM_001357943.2	126

**Table 2:** Demographic characteristics and laboratory findings of the study population.

Parameter	HCC (*n* = 53)	Control (*n* = 44)	*p*-Value
Age	55.43 ± 1.870	50.53 ± 1.709	0.06
Gender			0.819
Male	40 (75.47%)	25(56.8%)	
Female	13 (24.53%)	19 (43.2%)	
AST ^a^ (IU/L)	197 ± 34.04	79.8 ± 41.97	0.039 *
ALT ^b^ (IU/L)	93.53 ± 17.04	41.72 ± 10.26	0.0407 *
ALP ^c^ (IU/L)	565.8 ± 107.0	264.6 ± 38.42	0.05 *
Triglyceride (mg/dL)	107.7 ± 31.0	141.8 ± 30.78	0.459
Cholesterol (mg/dL)	199.5 ± 61.87	180 ± 18.64	0.7882
HDL ^d^ (mg/dL)	41.83 ± 6.237	36.5 ± 7.762	0.6055
LDL ^e^ (mg/dL)	171.4 ± 77.95	93.67 ± 21.88	0.4877
PLT ^f^ (×1000/mm^3^)	172.4 ± 13.11	216.8 ± 24.11	0.0842

Quantitative variables presented as mean ± SEM. *: significance value. ^a^: aspartate aminotransferase. ^b^: alanine aminotransferase. ^c^: alkaline phosphatase. ^d^: high-density lipoprotein. ^e^: low-density lipoprotein. ^f^: platelet.

**Table 3:** Distribution of histological grade (A) and disease stage (B) in HCC patients.

Parameter	PhaseI (%)	PhaseII (%)	PhaseIII (%)	PhaseIV (%)
Tumor grade	35.8	35.9	20.75	7.55
Clinical stage	60.41	8.33	4.2	27.06

**Table 4:** Effect of HBV-, HCV-related HCC on gene expression and liver function activity.

Parameter	HBV-Related HCC			HCV-Related HCC		
	HBV-Positive (*n* = 17)	HBV-Negative (*n* = 26)	*p*-Value	HCV-Positive (*n* = 7)	HCV-Negative (*n* = 36)	*p*-Value
SOX2	2.608 ± 3.049	2.751± 2.664	0.9	1.744± 1.539	2.882 ± 2.911	0.4
Bcl-2	5.703 ± 4.849	3.336 ± 2.220	0.4	6.162 ± 4.845	3.482 ± 2.649	0.1
Bax	0.284 ± 0.237	0.501 ± 0.430	0.3	0.705 ± 0.371	0.371 ± 0.380	0.2
AST ^a^	218.3 ± 193.4	190.5 ± 205.9	0.7	238.3 ± 212.0	208.5 ± 210.4	0.5
ALT ^b^	87.80 ± 75.71	96.65 ± 113.6	0.8	69.50 ± 34.35	97.69 ± 106.3	0.7
ALP ^c^	696.9 ± 897.7	466.7 ± 242.0	0.7	722.2 ± 368.5	532.9 ± 654.4	0.07
AFP ^d^	415.7 ± 427.2	140.5 ± 204.2	0.1	195.0 ± 186.5	4906 ± 21244	0.5
Survival	9.308± 6.762	9.167 ± 8.140	0.8	8.500 ± 5.802	8.962 ± 7.681	0.8

All data are presented as mean ± SD. ^a^: aspartate aminotransferase. ^b^: alanine aminotransferase. ^c^: alkaline phosphatase. ^d^: alpha-fetoprotein.

**Table 5:** Correlation between Bax, Bcl-2, and serum liver enzyme activity.

Correlation	Relative Expression of Bax		Relative Expression of Bcl-2	
	*p*-Value	Correlation Coefficient	*p*-Value	Correlation Coefficient
AST	0.1	−0.384	0.1	0.326
ALT	0.5	−0.187	0.02 *	0.486
AFP	0.03 *	0.593	0.1	0.320
ALP	0.3	−0.291	0.01 *	0.537

*: significance value.

## Data Availability

The datasets generated during and/or analyzed during the current study are available from the corresponding author on reasonable request.
